# Crystal structure of (aceto­nitrile-κ*N*)iodido­(2-(naphthalen-1-yl)-6-{1-[(2,4,6-tri­methyl­phen­yl)imino]ethyl}­pyridine-κ^2^ *N*,*N*′)copper(I)

**DOI:** 10.1107/S2056989016018685

**Published:** 2016-11-29

**Authors:** Nada Al-Najjar, Gregory A. Solan, Kuldip Singh

**Affiliations:** a Department of Chemistry, College of Science for Women, University of Baghdad, Iraq; b Department of Chemistry, University of Leicester, University Road, Leicester LE1 7RH, England

**Keywords:** crystal structure, copper(I) complex, 2-imino-6-(naphthalen-1-yl)pyridine, Schiff base, iodide, bidentate ligand

## Abstract

In the mononuclear title complex, [CuI(C_2_H_3_N)(C_26_H_24_N_2_)], the Cu^I^ ion has a distorted tetra­hedral coordination environment, defined by two N atoms of the chelating 2-(naphthalen-1-yl)-6-[(2,4,6-tri­methyl­phen­yl)imino]­pyridine ligand, one N atom of an aceto­nitrile ligand and one iodide ligand. Within the complex, there are weak intra­molecular C—H⋯N hydrogen bonds, while weak inter­molecular C—H⋯I inter­actions between complex mol­ecules, help to facilitate a three-dimensional network.

## Abstract 2

The Cu^I^ atom in the mol­ecular structure of the title compound exhibits a distorted tetra­hedral coordination sphere, defined by two N atoms of the chelating ligand, one N atom of the aceto­nitrile ligand and one iodide ligand.

## Chemical context   

Coordination complexes of copper(I) halides bearing a variety of co-ligands have been of inter­est in coordination chemistry (Karahan *et al.*, 2015 ▸; Dennehy *et al.*, 2011 ▸; Oshio *et al.*, 1996 ▸; Seward *et al.*, 2003 ▸) due, in some measure, to their preparative accessibility, structural variability, magnetic properties (Oshio *et al.*, 1996 ▸) and their relevance to biological or medicinal applications (Corey *et al.*, 1987 ▸; Dias *et al.*, 2006 ▸). The role of copper(I) is evident in several biologically important reactions, such as a di­oxy­gen carrier and models for several enzymes (Krupanidhi *et al.*, 2008 ▸). Elsewhere, these compounds have been reported to be luminescent (Aslanidis *et al.*, 2010 ▸; Gallego *et al.*, 2012 ▸) and exhibit corrosion inhibit­ing properties (Tian *et al.*, 2004 ▸). The structures of metal complexes bearing naphthyl-substituted *N*,*N*-pyridine-alkyl­amides were reported by Armitage *et al.* (2015 ▸) and related structures were presented by Wattanakanjana *et al.* (2014 ▸). Cotton *et al.* (1999 ▸) highlighted details of the affinity of nitrile ligands for Cu^I^ ions. Within this context, we report herein the crystal structure of the title complex, [CuI(C_2_H_3_N)(C_26_H_24_N_2_)].
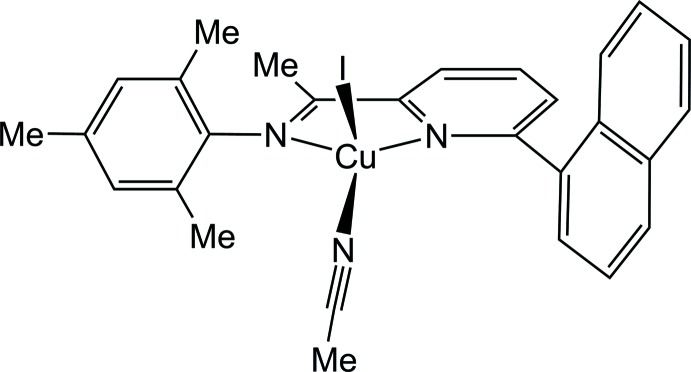


## Structural commentary   

**Figure 1:**
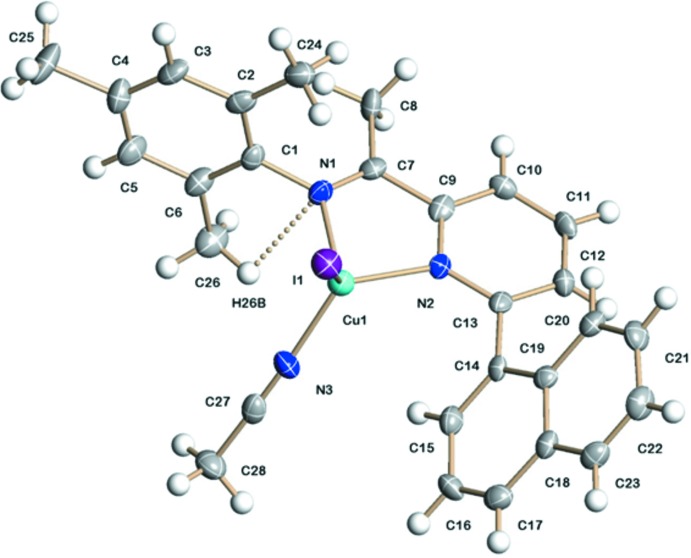
The mol­ecular structure of the title complex, with displacement ellipsoids drawn at the 50% probability level. The C—H⋯N hydrogen bond is shown as a dashed line.

The mol­ecular structure of the title complex is shown in Fig. 1 ▸. The Cu^I^ ion is coordinated by atoms N1 and N2 of the 2-(naphthalen-1-yl)-6-[(2,4,6-tri­methyl­phen­yl)imino]­pyridine ligand, by atom N3 of an aceto­nitrile ligand and by an iodide anion (I1), leading to a distorted tetra­hedral coordination environment. The two N atoms of the bidentate ligand chelate to Cu^I^ with similar Cu—N bond lengths [Cu1—N1 = 2.091 (4), Cu1—N2 = 2.085 (4) Å]. A comparable *N*,*N*′-binding has been observed in related structures with bis­[2-(2-pyrid­yl)eth­yl]amine ligands (Osako *et al.*, 2001 ▸). At 1.960 (5) Å, the Cu1—N3 distance is significantly shorter than the Cu—N_pyridine_ and Cu—N_imine_ distances. The Cu1—I distance amounts to 2.5479 (9) Å. The N2—Cu1—N1 bite angle of the chelating ligand is 78.86 (18)°, while the N3—Cu—I angle between the monodentate aceto­nitrile and iodide ligands is closer to tetra­hedral, 112.74 (15)°. The naphthyl ring system is inclined by 58.20 (17)° to the central N=C(CH_3_)—pyridine moiety, whereas the tri­methyl­phenyl ring is almost perpendicular to the latter, at 84.8 (3)°. Within the complex, an intra­molecular C—H⋯N hydrogen-bonding inter­action is present, stabil­izing the mol­ecular conformation (Table 1 ▸, Fig. 1 ▸).

## Supra­molecular features   

**Figure 2:**
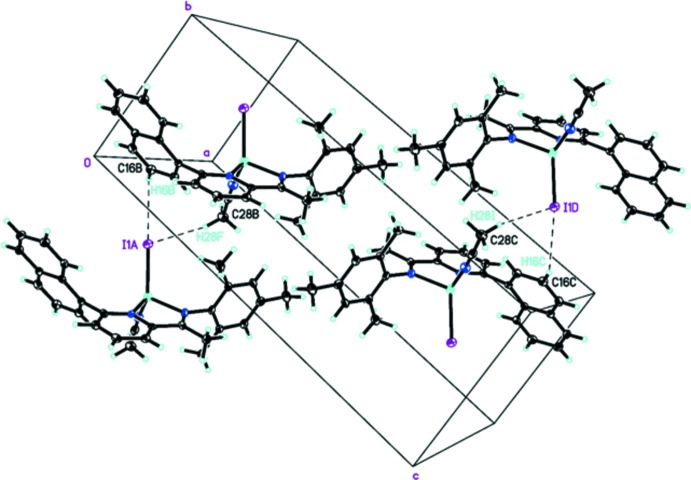
Part of the crystal structure, showing inter­molecular C—H⋯I inter­actions (dashed lines).

In the crystal, weak C—H⋯I contacts involving a phenyl H atom [C16—H16*B*⋯I^i^, 3.958 (6) Å, 152°; symmetry code: (i) *x*, *y* − 1, *z*] and a H atom of the aceto­nitrile methyl group [[C28—H28*B*⋯I^i^, 4.010 (6) Å, 109°] link the complex mol­ecules, forming a three-dimensional network (Fig. 2 ▸).

## Synthesis and crystallization   

All synthetic manipulations were performed under a nitro­gen atmosphere, using standard Schlenk techniques. Solvents were distilled under nitro­gen from appropriate drying agents and degassed prior to use (Armarego *et al.*, 1996 ▸). The 2-(naphthalen-1-yl)-6-[(2,4,6-tri­methyl­phen­yl)imino]­pyridine ligand (*L*_mes_) was synthesized according to a modified literature procedure (Armitage *et al.*, 2015 ▸).

A solution of 0.0262 g of CuI (0.137 mmol) in 5 ml of aceto­nitrile was mixed with a solution of 0.05 g of *L*_mes_ (0.134 mmol) in 5 ml of aceto­nitrile. The mixture was stirred at room temperature for 24 h before evaporating the volatiles. The residue was extracted with *n*-hexane (5 × 3 ml). The extracts were combined and the solvent removed under reduced pressure to give a red solid which was recrystallized from aceto­nitrile solution. Yield: 54%. M.p. >253 K (decomp). ^1^H NMR (400 MHz, CD_2_Cl_2_): δ 1.88 [*s*, 6H, *ortho*- (CH_3_)_2_], 1.97 (*s*, 3H, N≡CCH_3_), 2.16 (*s*, 3H, N=CCH_3_), 2.20 [*s*, 3H, *para*-(CH_3_)_2_], 6.84 (*s*, 2H, Mes-H), 7.39 (*s*, 1H, Nap-H), 7.45 (*t*, *J* 7.8, 2H, Nap-H/Py–H), 7.51 (*s*, 1H, Py–H), 7.73 (*s*, 1H, Py-H), 7.81 (*s*, 2H, Nap-H), 7.87 (*d*, *J* 3.7, 2H, Nap-H), 8.04 (*s*, 1H, Nap-H). IR ν_max_ (solid)/cm^−1^ 1620 (C=N_imine_), 1555 (C=N_py_). ESI MS: *m*/*z* 428 [*M*–I–MeCN]^+^.

## Refinement   

Crystal data, data collection and structure refinement details are summarized in Table 2 ▸. Hydrogen atoms were positioned geometrically, with C—H = 0.95 Å and with *U*_iso_(H) = 1.2*U*_eq_(C) for H atoms on C*sp* ^2^ and 0.98 Å with *U*_iso_(H) = 1.5*U*_eq_(C) for H atoms on C*sp* ^3^.

## Supplementary Material

Crystal structure: contains datablock(s) I. DOI: 10.1107/S2056989016018685/wm5341sup1.cif

Structure factors: contains datablock(s) I. DOI: 10.1107/S2056989016018685/wm5341Isup2.hkl

CCDC reference: 1518571

Additional supporting information: crystallographic information; 3D view; checkCIF report

## Figures and Tables

**Table 1:** Hydrogen-bond geometry (Å, °)

*D*—H⋯*A*	*D*—H	H⋯*A*	*D*⋯*A*	*D*—H⋯*A*
C26—H26*B*⋯N1	0.98	2.52	2.891 (8)	102

**Table 2:** Experimental details

Crystal data	
Chemical formula	[CuI(C_2_H_3_N)(C_26_H_24_N_2_)]
*M*_r_	595.97
Crystal system, space group	Monoclinic, *P*2_1_/*c*
Temperature (K)	150
*a*, *b*, *c* (Å)	14.689 (3), 8.0775 (15), 21.861 (4)
β (°)	103.942 (3)
*V* (Å^3^)	2517.4 (8)
*Z*	4
Radiation type	Mo *K*α
μ (mm^−1^)	2.11
Crystal size (mm)	0.25 × 0.07 × 0.03

Data collection	
Diffractometer	Bruker APEX 2000 CCD area detector
Absorption correction	Multi-scan (*SADABS*; Bruker, 2001 ▸)
*T*_min_, *T*_max_	0.679, 0.862
No. of measured, independent and observed [*I* > 2σ(*I*)] reflections	19143, 4936, 2757
*R*_int_	0.125
(sin θ/λ)_max_ (Å^−1^)	0.617

Refinement	
*R*[*F* ^2^ > 2σ(*F* ^2^)], *wR*(*F* ^2^), *S*	0.051, 0.085, 0.77
No. of reflections	4936
No. of parameters	303
H-atom treatment	H-atom parameters constrained
Δρ_max_, Δρ_min_ (e Å^−3^)	1.13, −0.81

Computer programs: *SMART* and *SAINT* (Bruker, 2001 ▸), *SHELXS97*, *SHELXL97* and *SHELXTL* (Sheldrick, 2008 ▸).

## supplementary crystallographic information

### Crystal data

**Table:**

[CuI(C_2_H_3_N)(C_26_H_24_N_2_)]	*F*(000) = 1192
*M**_r_* = 595.97	*D*_x_ = 1.572 Mg m^−^^3^
Monoclinic, *P*2_1_/*c*	Mo *K*α radiation, λ = 0.71073 Å
Hall symbol: -P 2ybc	Cell parameters from 639 reflections
*a* = 14.689 (3) Å	θ = 2.9–23.2°
*b* = 8.0775 (15) Å	µ = 2.11 mm^−^^1^
*c* = 21.861 (4) Å	*T* = 150 K
β = 103.942 (3)°	Needle, orange
*V* = 2517.4 (8) Å^3^	0.25 × 0.07 × 0.03 mm
*Z* = 4	

### Data collection

**Table:**

Bruker APEX 2000 CCD area detector diffractometer	4936 independent reflections
Radiation source: fine-focus sealed tube	2757 reflections with *I* > 2σ(*I*)
Graphite monochromator	*R*_int_ = 0.125
phi and ω scans	θ_max_ = 26.0°, θ_min_ = 1.4°
Absorption correction: multi-scan (*SADABS*; Bruker, 2001)	*h* = −18→17
*T*_min_ = 0.679, *T*_max_ = 0.862	*k* = −9→9
19143 measured reflections	*l* = −26→26

### Refinement

**Table:**

Refinement on *F*^2^	Primary atom site location: structure-invariant direct methods
Least-squares matrix: full	Secondary atom site location: difference Fourier map
*R*[*F*^2^ > 2σ(*F*^2^)] = 0.051	Hydrogen site location: inferred from neighbouring sites
*wR*(*F*^2^) = 0.085	H-atom parameters constrained
*S* = 0.77	*w* = 1/[σ^2^(*F*_o_^2^) + (0.0178*P*)^2^] where *P* = (*F*_o_^2^ + 2*F*_c_^2^)/3
4936 reflections	(Δ/σ)_max_ = 0.002
303 parameters	Δρ_max_ = 1.13 e Å^−^^3^
0 restraints	Δρ_min_ = −0.81 e Å^−^^3^

### Special details

**Table:**

Geometry. All esds (except the esd in the dihedral angle between two l.s. planes) are estimated using the full covariance matrix. The cell esds are taken into account individually in the estimation of esds in distances, angles and torsion angles; correlations between esds in cell parameters are only used when they are defined by crystal symmetry. An approximate (isotropic) treatment of cell esds is used for estimating esds involving l.s. planes.
Refinement. Refinement of F^2^ against ALL reflections. The weighted R-factor wR and goodness of fit S are based on F^2^, conventional R-factors R are based on F, with F set to zero for negative F^2^. The threshold expression of F^2^ > 2sigma(F^2^) is used only for calculating R-factors(gt) etc. and is not relevant to the choice of reflections for refinement. R-factors based on F^2^ are statistically about twice as large as those based on F, and R- factors based on ALL data will be even larger.

### Fractional atomic coordinates and isotropic or equivalent isotropic displacement parameters (Å^2^)

**Table:**

	*x*	*y*	*z*	*U*_iso_*/*U*_eq_	
Cu1	0.27041 (5)	0.46133 (9)	0.24724 (3)	0.0280 (2)	
I1	0.27321 (3)	0.69329 (5)	0.16873 (2)	0.03325 (13)	
N1	0.2948 (3)	0.5389 (6)	0.3410 (2)	0.0241 (12)	
N2	0.1440 (3)	0.3972 (5)	0.2685 (2)	0.0196 (11)	
N3	0.3532 (3)	0.2768 (6)	0.2390 (2)	0.0307 (13)	
C1	0.3799 (4)	0.6189 (7)	0.3735 (3)	0.0241 (14)	
C2	0.3847 (4)	0.7897 (8)	0.3723 (3)	0.0277 (15)	
C3	0.4709 (4)	0.8651 (7)	0.3999 (3)	0.0309 (16)	
H3	0.4755	0.9824	0.3996	0.037*	
C4	0.5483 (4)	0.7737 (8)	0.4270 (3)	0.0322 (16)	
C5	0.5416 (4)	0.6047 (8)	0.4277 (3)	0.0335 (16)	
H5	0.5956	0.5412	0.4463	0.040*	
C6	0.4569 (4)	0.5231 (7)	0.4015 (3)	0.0293 (15)	
C7	0.2277 (4)	0.5153 (7)	0.3683 (3)	0.0245 (14)	
C8	0.2289 (4)	0.5595 (7)	0.4358 (2)	0.0293 (15)	
H8A	0.2892	0.6104	0.4560	0.044*	
H8B	0.2200	0.4590	0.4588	0.044*	
H8C	0.1781	0.6378	0.4363	0.044*	
C9	0.1407 (4)	0.4362 (7)	0.3290 (3)	0.0237 (14)	
C10	0.0617 (4)	0.4098 (7)	0.3511 (3)	0.0246 (14)	
H10	0.0612	0.4389	0.3931	0.030*	
C11	−0.0174 (4)	0.3404 (7)	0.3115 (3)	0.0260 (15)	
H11	−0.0723	0.3197	0.3260	0.031*	
C12	−0.0143 (4)	0.3028 (7)	0.2511 (3)	0.0249 (14)	
H12	−0.0679	0.2569	0.2230	0.030*	
C13	0.0668 (4)	0.3312 (7)	0.2308 (3)	0.0224 (14)	
C14	0.0726 (4)	0.2803 (7)	0.1655 (3)	0.0193 (13)	
C15	0.1401 (4)	0.1717 (7)	0.1593 (3)	0.0281 (15)	
H15	0.1865	0.1391	0.1956	0.034*	
C16	0.1429 (4)	0.1057 (7)	0.0993 (3)	0.0282 (15)	
H16	0.1905	0.0295	0.0955	0.034*	
C17	0.0766 (4)	0.1533 (7)	0.0478 (3)	0.0303 (16)	
H17	0.0781	0.1087	0.0079	0.036*	
C18	0.0056 (4)	0.2669 (7)	0.0517 (3)	0.0244 (15)	
C19	0.0043 (4)	0.3361 (7)	0.1116 (3)	0.0224 (14)	
C20	−0.0648 (4)	0.4581 (7)	0.1131 (3)	0.0266 (15)	
H20	−0.0671	0.5070	0.1522	0.032*	
C21	−0.1273 (4)	0.5060 (7)	0.0600 (3)	0.0305 (16)	
H21	−0.1712	0.5906	0.0626	0.037*	
C22	−0.1293 (4)	0.4334 (8)	0.0006 (3)	0.0337 (16)	
H22	−0.1754	0.4644	−0.0361	0.040*	
C23	−0.0625 (4)	0.3175 (7)	−0.0021 (3)	0.0294 (15)	
H23	−0.0620	0.2693	−0.0417	0.035*	
C24	0.3003 (4)	0.8937 (7)	0.3427 (3)	0.0347 (17)	
H24A	0.2747	0.8572	0.2992	0.052*	
H24B	0.3189	1.0102	0.3428	0.052*	
H24C	0.2524	0.8811	0.3668	0.052*	
C25	0.6418 (4)	0.8592 (8)	0.4547 (3)	0.051 (2)	
H25A	0.6356	0.9314	0.4895	0.076*	
H25B	0.6598	0.9255	0.4220	0.076*	
H25C	0.6901	0.7755	0.4704	0.076*	
C26	0.4524 (4)	0.3376 (7)	0.4039 (3)	0.0379 (17)	
H26A	0.5139	0.2910	0.4037	0.057*	
H26B	0.4055	0.2970	0.3672	0.057*	
H26C	0.4347	0.3036	0.4426	0.057*	
C27	0.4029 (4)	0.1710 (8)	0.2387 (3)	0.0284 (15)	
C28	0.4682 (4)	0.0349 (7)	0.2396 (3)	0.0367 (17)	
H28A	0.5068	0.0200	0.2825	0.055*	
H28B	0.5088	0.0601	0.2112	0.055*	
H28C	0.4330	−0.0670	0.2257	0.055*	

### Atomic displacement parameters (Å^2^)

**Table:**

	*U*^11^	*U*^22^	*U*^33^	*U*^12^	*U*^13^	*U*^23^
Cu1	0.0280 (4)	0.0304 (5)	0.0269 (4)	−0.0010 (4)	0.0089 (4)	−0.0029 (4)
I1	0.0350 (2)	0.0339 (3)	0.0303 (2)	0.0026 (2)	0.00674 (18)	0.0044 (2)
N1	0.024 (3)	0.027 (3)	0.021 (3)	−0.003 (2)	0.002 (2)	−0.003 (2)
N2	0.018 (3)	0.021 (3)	0.018 (3)	0.001 (2)	0.000 (2)	0.003 (2)
N3	0.025 (3)	0.033 (3)	0.037 (3)	0.004 (3)	0.012 (3)	−0.002 (3)
C1	0.022 (4)	0.032 (4)	0.017 (3)	−0.005 (3)	0.002 (3)	−0.002 (3)
C2	0.038 (4)	0.029 (4)	0.017 (3)	−0.007 (3)	0.009 (3)	−0.006 (3)
C3	0.040 (4)	0.025 (4)	0.028 (4)	−0.013 (3)	0.008 (3)	−0.006 (3)
C4	0.024 (4)	0.042 (5)	0.028 (4)	−0.008 (3)	0.001 (3)	0.007 (3)
C5	0.033 (4)	0.033 (4)	0.028 (4)	−0.004 (3)	−0.004 (3)	−0.001 (3)
C6	0.036 (4)	0.028 (4)	0.022 (4)	−0.009 (3)	0.003 (3)	0.002 (3)
C7	0.029 (4)	0.018 (3)	0.025 (4)	−0.003 (3)	0.005 (3)	−0.003 (3)
C8	0.018 (3)	0.039 (4)	0.030 (4)	−0.008 (3)	0.005 (3)	0.002 (3)
C9	0.027 (4)	0.015 (3)	0.027 (4)	−0.001 (3)	0.003 (3)	0.007 (3)
C10	0.029 (4)	0.024 (4)	0.024 (4)	0.004 (3)	0.012 (3)	0.002 (3)
C11	0.021 (3)	0.024 (4)	0.037 (4)	−0.001 (3)	0.016 (3)	0.002 (3)
C12	0.018 (3)	0.028 (4)	0.029 (4)	−0.001 (3)	0.006 (3)	0.001 (3)
C13	0.021 (3)	0.014 (3)	0.030 (4)	0.002 (3)	0.002 (3)	−0.002 (3)
C14	0.014 (3)	0.021 (3)	0.023 (3)	−0.005 (3)	0.004 (3)	0.003 (3)
C15	0.022 (3)	0.027 (4)	0.033 (4)	0.000 (3)	0.001 (3)	0.003 (3)
C16	0.026 (4)	0.029 (4)	0.034 (4)	0.005 (3)	0.014 (3)	0.000 (3)
C17	0.034 (4)	0.028 (4)	0.031 (4)	−0.006 (3)	0.011 (3)	−0.005 (3)
C18	0.021 (3)	0.025 (4)	0.026 (4)	−0.006 (3)	0.002 (3)	0.003 (3)
C19	0.024 (3)	0.018 (4)	0.025 (3)	−0.004 (3)	0.006 (3)	−0.001 (3)
C20	0.027 (4)	0.024 (4)	0.026 (4)	−0.007 (3)	0.003 (3)	−0.002 (3)
C21	0.023 (4)	0.029 (4)	0.038 (4)	0.007 (3)	0.004 (3)	0.004 (3)
C22	0.031 (4)	0.036 (4)	0.029 (4)	−0.001 (3)	−0.001 (3)	0.008 (3)
C23	0.034 (4)	0.034 (4)	0.020 (3)	−0.008 (3)	0.007 (3)	−0.001 (3)
C24	0.038 (4)	0.032 (4)	0.037 (4)	−0.004 (3)	0.014 (3)	−0.008 (3)
C25	0.041 (4)	0.047 (5)	0.054 (5)	−0.024 (4)	−0.008 (4)	0.001 (4)
C26	0.042 (4)	0.032 (4)	0.034 (4)	0.001 (3)	−0.002 (3)	0.007 (3)
C27	0.024 (4)	0.040 (4)	0.022 (3)	−0.006 (3)	0.006 (3)	0.000 (3)
C28	0.035 (4)	0.031 (4)	0.046 (4)	0.006 (3)	0.014 (3)	0.000 (3)

### Geometric parameters (Å, º)

**Table:**

Cu1—N3	1.960 (5)	C13—C14	1.507 (7)
Cu1—N2	2.085 (4)	C14—C15	1.355 (7)
Cu1—N1	2.091 (4)	C14—C19	1.426 (7)
Cu1—I1	2.5479 (9)	C15—C16	1.427 (7)
N1—C7	1.282 (7)	C15—H15	0.9500
N1—C1	1.434 (6)	C16—C17	1.354 (7)
N2—C13	1.342 (6)	C16—H16	0.9500
N2—C9	1.372 (6)	C17—C18	1.408 (7)
N3—C27	1.125 (7)	C17—H17	0.9500
C1—C2	1.382 (7)	C18—C23	1.409 (7)
C1—C6	1.386 (7)	C18—C19	1.427 (7)
C2—C3	1.403 (7)	C19—C20	1.420 (7)
C2—C24	1.508 (7)	C20—C21	1.351 (7)
C3—C4	1.366 (8)	C20—H20	0.9500
C3—H3	0.9500	C21—C22	1.420 (8)
C4—C5	1.369 (8)	C21—H21	0.9500
C4—C25	1.527 (7)	C22—C23	1.368 (7)
C5—C6	1.402 (7)	C22—H22	0.9500
C5—H5	0.9500	C23—H23	0.9500
C6—C26	1.501 (7)	C24—H24A	0.9800
C7—C9	1.500 (7)	C24—H24B	0.9800
C7—C8	1.515 (7)	C24—H24C	0.9800
C8—H8A	0.9800	C25—H25A	0.9800
C8—H8B	0.9800	C25—H25B	0.9800
C8—H8C	0.9800	C25—H25C	0.9800
C9—C10	1.377 (7)	C26—H26A	0.9800
C10—C11	1.389 (7)	C26—H26B	0.9800
C10—H10	0.9500	C26—H26C	0.9800
C11—C12	1.364 (7)	C27—C28	1.456 (8)
C11—H11	0.9500	C28—H28A	0.9800
C12—C13	1.387 (7)	C28—H28B	0.9800
C12—H12	0.9500	C28—H28C	0.9800
			
N3—Cu1—N2	115.94 (19)	C15—C14—C19	120.5 (5)
N3—Cu1—N1	110.75 (19)	C15—C14—C13	118.8 (5)
N2—Cu1—N1	78.86 (18)	C19—C14—C13	120.5 (5)
N3—Cu1—I1	112.74 (15)	C14—C15—C16	121.2 (5)
N2—Cu1—I1	119.51 (12)	C14—C15—H15	119.4
N1—Cu1—I1	114.43 (13)	C16—C15—H15	119.4
C7—N1—C1	120.8 (5)	C17—C16—C15	118.9 (6)
C7—N1—Cu1	116.2 (4)	C17—C16—H16	120.5
C1—N1—Cu1	122.9 (4)	C15—C16—H16	120.5
C13—N2—C9	117.5 (5)	C16—C17—C18	122.0 (6)
C13—N2—Cu1	128.9 (4)	C16—C17—H17	119.0
C9—N2—Cu1	113.5 (4)	C18—C17—H17	119.0
C27—N3—Cu1	175.3 (5)	C17—C18—C23	121.7 (6)
C2—C1—C6	121.7 (6)	C17—C18—C19	119.0 (5)
C2—C1—N1	118.9 (5)	C23—C18—C19	119.2 (5)
C6—C1—N1	119.2 (5)	C20—C19—C14	124.3 (5)
C1—C2—C3	118.0 (6)	C20—C19—C18	117.5 (5)
C1—C2—C24	121.6 (5)	C14—C19—C18	118.2 (5)
C3—C2—C24	120.4 (6)	C21—C20—C19	121.4 (6)
C4—C3—C2	121.5 (6)	C21—C20—H20	119.3
C4—C3—H3	119.2	C19—C20—H20	119.3
C2—C3—H3	119.2	C20—C21—C22	121.8 (6)
C3—C4—C5	119.3 (6)	C20—C21—H21	119.1
C3—C4—C25	120.2 (6)	C22—C21—H21	119.1
C5—C4—C25	120.5 (6)	C23—C22—C21	117.8 (6)
C4—C5—C6	121.6 (6)	C23—C22—H22	121.1
C4—C5—H5	119.2	C21—C22—H22	121.1
C6—C5—H5	119.2	C22—C23—C18	122.3 (6)
C1—C6—C5	117.9 (6)	C22—C23—H23	118.8
C1—C6—C26	122.3 (6)	C18—C23—H23	118.8
C5—C6—C26	119.8 (6)	C2—C24—H24A	109.5
N1—C7—C9	116.2 (5)	C2—C24—H24B	109.5
N1—C7—C8	126.0 (5)	H24A—C24—H24B	109.5
C9—C7—C8	117.8 (5)	C2—C24—H24C	109.5
C7—C8—H8A	109.5	H24A—C24—H24C	109.5
C7—C8—H8B	109.5	H24B—C24—H24C	109.5
H8A—C8—H8B	109.5	C4—C25—H25A	109.5
C7—C8—H8C	109.5	C4—C25—H25B	109.5
H8A—C8—H8C	109.5	H25A—C25—H25B	109.5
H8B—C8—H8C	109.5	C4—C25—H25C	109.5
N2—C9—C10	122.0 (5)	H25A—C25—H25C	109.5
N2—C9—C7	115.2 (5)	H25B—C25—H25C	109.5
C10—C9—C7	122.7 (5)	C6—C26—H26A	109.5
C9—C10—C11	119.6 (5)	C6—C26—H26B	109.5
C9—C10—H10	120.2	H26A—C26—H26B	109.5
C11—C10—H10	120.2	C6—C26—H26C	109.5
C12—C11—C10	118.3 (5)	H26A—C26—H26C	109.5
C12—C11—H11	120.8	H26B—C26—H26C	109.5
C10—C11—H11	120.8	N3—C27—C28	178.7 (7)
C11—C12—C13	120.2 (5)	C27—C28—H28A	109.5
C11—C12—H12	119.9	C27—C28—H28B	109.5
C13—C12—H12	119.9	H28A—C28—H28B	109.5
N2—C13—C12	122.2 (5)	C27—C28—H28C	109.5
N2—C13—C14	117.2 (5)	H28A—C28—H28C	109.5
C12—C13—C14	120.5 (5)	H28B—C28—H28C	109.5
			
N3—Cu1—N1—C7	−113.7 (4)	N1—C7—C9—N2	0.5 (7)
N2—Cu1—N1—C7	0.1 (4)	C8—C7—C9—N2	−179.3 (5)
I1—Cu1—N1—C7	117.6 (4)	N1—C7—C9—C10	−177.1 (5)
N3—Cu1—N1—C1	68.3 (5)	C8—C7—C9—C10	3.0 (8)
N2—Cu1—N1—C1	−178.0 (5)	N2—C9—C10—C11	0.6 (8)
I1—Cu1—N1—C1	−60.5 (4)	C7—C9—C10—C11	178.1 (5)
N3—Cu1—N2—C13	−74.9 (5)	C9—C10—C11—C12	−1.0 (8)
N1—Cu1—N2—C13	177.3 (5)	C10—C11—C12—C13	1.0 (9)
I1—Cu1—N2—C13	65.3 (5)	C9—N2—C13—C12	0.2 (8)
N3—Cu1—N2—C9	108.0 (4)	Cu1—N2—C13—C12	−176.7 (4)
N1—Cu1—N2—C9	0.2 (4)	C9—N2—C13—C14	−176.8 (5)
I1—Cu1—N2—C9	−111.7 (3)	Cu1—N2—C13—C14	6.3 (7)
C7—N1—C1—C2	−86.8 (7)	C11—C12—C13—N2	−0.7 (9)
Cu1—N1—C1—C2	91.2 (6)	C11—C12—C13—C14	176.2 (5)
C7—N1—C1—C6	97.6 (7)	N2—C13—C14—C15	56.3 (7)
Cu1—N1—C1—C6	−84.4 (6)	C12—C13—C14—C15	−120.8 (6)
C6—C1—C2—C3	0.7 (9)	N2—C13—C14—C19	−128.1 (5)
N1—C1—C2—C3	−174.8 (5)	C12—C13—C14—C19	54.8 (8)
C6—C1—C2—C24	−179.1 (5)	C19—C14—C15—C16	−2.4 (8)
N1—C1—C2—C24	5.4 (8)	C13—C14—C15—C16	173.3 (5)
C1—C2—C3—C4	0.2 (9)	C14—C15—C16—C17	0.1 (9)
C24—C2—C3—C4	180.0 (5)	C15—C16—C17—C18	0.5 (9)
C2—C3—C4—C5	−0.3 (9)	C16—C17—C18—C23	179.2 (6)
C2—C3—C4—C25	178.0 (5)	C16—C17—C18—C19	1.1 (8)
C3—C4—C5—C6	−0.5 (10)	C15—C14—C19—C20	−175.4 (5)
C25—C4—C5—C6	−178.8 (5)	C13—C14—C19—C20	9.0 (8)
C2—C1—C6—C5	−1.4 (9)	C15—C14—C19—C18	3.9 (8)
N1—C1—C6—C5	174.1 (5)	C13—C14—C19—C18	−171.7 (5)
C2—C1—C6—C26	179.1 (5)	C17—C18—C19—C20	176.1 (5)
N1—C1—C6—C26	−5.4 (9)	C23—C18—C19—C20	−2.1 (8)
C4—C5—C6—C1	1.3 (9)	C17—C18—C19—C14	−3.2 (8)
C4—C5—C6—C26	−179.2 (6)	C23—C18—C19—C14	178.6 (5)
C1—N1—C7—C9	177.8 (5)	C14—C19—C20—C21	179.7 (5)
Cu1—N1—C7—C9	−0.3 (7)	C18—C19—C20—C21	0.5 (8)
C1—N1—C7—C8	−2.4 (9)	C19—C20—C21—C22	2.1 (9)
Cu1—N1—C7—C8	179.5 (4)	C20—C21—C22—C23	−3.0 (9)
C13—N2—C9—C10	−0.2 (8)	C21—C22—C23—C18	1.3 (9)
Cu1—N2—C9—C10	177.2 (4)	C17—C18—C23—C22	−176.9 (5)
C13—N2—C9—C7	−177.8 (5)	C19—C18—C23—C22	1.2 (9)
Cu1—N2—C9—C7	−0.5 (6)		

### Hydrogen-bond geometry (Å, º)

**Table:**

*D*—H···*A*	*D*—H	H···*A*	*D*···*A*	*D*—H···*A*
C26—H26*B*···N1	0.98	2.52	2.891 (8)	102
